# Aligning Measurement, Scoring, and Interpretation in Survey‐Based NSSI Research

**DOI:** 10.1002/npr2.70118

**Published:** 2026-04-07

**Authors:** Kenjiro Shiraishi

**Affiliations:** ^1^ Tanashi Kitaguchi Acupuncture and Moxa Clinic Tokyo Japan

## Abstract

This graphical abstract highlights the central interpretive point of the Letter: the survey measured public views about possible functions of NSSI, not the actual motives of people who engage in NSSI. Clearer alignment between measurement target, scoring, and inference may improve reproducibility and interpretation.

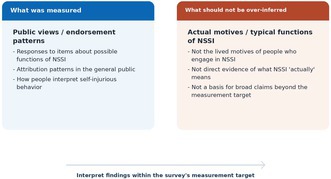

Dear Editor,

I read with great interest the article by Takahashi et al. which examined how the Japanese public perceives the functions of non‐suicidal self‐injury (NSSI) using a large online survey, exploratory factor analysis (EFA), and two‐way analyses by gender and age group [1]. This is an important and timely topic, particularly from the standpoint of public education and stigma reduction. I respectfully offer several methodological clarifications that may help strengthen the article's reproducibility and sharpen the alignment between what was measured and what may reasonably be inferred from the findings.

First, the definition of the analyzed outcome appears to require clarification. The abstract refers to “factor scores” as dependent variables, whereas the Methods section states that the authors used the mean scores of items with high loadings on each factor as subscale scores. These are not equivalent quantities. Factor scores are model‐based estimates that depend on the factor solution and scoring method, whereas item‐mean composites are observed‐score summaries. If the analyses were conducted using item‐mean subscale scores, describing them as “factor scores” may unintentionally make it difficult for readers to understand precisely what was analyzed and how the results might be reproduced. It would therefore be helpful to specify, for each analysis, which score was entered and how it was constructed, including any weighting, reverse coding, or handling of missing values.

Second, the EFA procedure would benefit from somewhat fuller reporting of the decision process. The article notes that the number of factors was determined based on eigenvalue attenuation and interpretability, and that items were iteratively removed when loadings were below 0.40 or when cross‐loadings were present. These are understandable analytic choices in exploratory work. However, a more explicit decision log—for example, the operational definition of cross‐loading, the number of iterations performed, and the order in which items were excluded—would make it easier for readers to evaluate the robustness of the final four‐factor solution [2, 3]. In particular, the fourth factor, which is defined by two items, is not necessarily problematic in an exploratory analysis, but it does warrant especially careful reporting and cautious interpretation.

Third, I wonder whether the interpretation of the factor structure might be framed somewhat more closely to the measurement target of the survey itself. As presented in the Appendix of Takahashi et al. the 20 items were created to evaluate respondents' views regarding possible functions of NSSI [1]. In that sense, the study directly measures patterns of public endorsement or attribution. This is valuable in its own right. At the same time, some passages in the Discussion and Conclusion appear to move from public endorsement patterns toward broader statements about the actual reasons for NSSI or the functions of self‐injurious behavior more generally. This point may be especially relevant where the Discussion moves from public endorsement patterns to statements about the “actual reasons” for NSSI or that NSSI “typically functions” in a particular way [1]. A more cautious framing may help here: the present findings seem most directly informative about how members of the public interpret NSSI, including potentially stigmatizing attribution patterns, rather than about the motives of people who engage in NSSI themselves. Framing the findings more consistently in terms of public perception may therefore strengthen, rather than weaken, the educational implications of the study. If those implications are discussed in relation to stigma reduction, it may also be helpful to clarify which attribution patterns are being interpreted as potentially stigmatizing.

Relatedly, the article states that the 20 items were adapted from prior studies [1]. If so, one additional clarification that may be useful is a clearer item‐to‐source or item‐to‐domain mapping. Such reporting would help readers understand how each item relates to prior literature and to the conceptual domains the survey intended to assess. This point may also be viewed in light of broader COSMIN principles [4, 5, 6]. Although COSMIN was developed primarily for studies on the measurement properties of patient‐reported outcome measures, its emphasis on transparent reporting and content validity is relevant here as a general methodological orientation [4, 5, 6]. Clearer reporting of item provenance, construct mapping, and score construction would help readers judge whether the derived domains adequately reflect the constructs under discussion.

Finally, if space permits, the article might also briefly indicate how multiple testing was considered across the four factor‐based analyses, and encourage readers to interpret statistically significant findings alongside their effect sizes [1]. This would further support a balanced reading of the results, especially in a large sample.

In summary, I respectfully suggest that the article could be further strengthened by: (1) clarifying the distinction between factor scores and item‐mean subscale scores; (2) providing a more explicit EFA decision log; (3) reporting clearer item‐to‐source or item‐to‐domain mapping; and (4) aligning the scope of interpretation more closely with the survey's measurement target, namely public perceptions of NSSI functions. These refinements would, in my view, make the findings easier to reproduce and their educational implications easier to interpret with confidence.

Sincerely,

Kenjiro Shiraishi

## Funding

The author has nothing to report.

## Consent

The author has nothing to report.

## Conflicts of Interest

The author declares no conflicts of interest.

## Data Availability

Data sharing not applicable to this article as no datasets were generated or analysed during the current study.
